# Absolute Position Coding Method for Angular Sensor—Single-Track Gray Codes

**DOI:** 10.3390/s18082728

**Published:** 2018-08-19

**Authors:** Fan Zhang, Hengjun Zhu, Kan Bian, Pengcheng Liu, Jianhui Zhang

**Affiliations:** 1 School of Mechanical and Electrical Engineering, Guangzhou University, Guangzhou 510006, China; zhangfan1107@bjtu.edu.cn (F.Z.); bk@nuaa.edu.cn (K.B.); pch060710111@hotmail.com (P.L.); 2 School of Mechanical, Electronic and Control Engineering, Beijing Jiaotong University, Beijing 100044, China; hjzhu@bjtu.edu.cn; 3 State Key Laboratory of Mechanics and Control of Mechanical Structures, Nanjing University of Aeronautics and Astronautics, Nanjing 210016, China

**Keywords:** absolute position sensing, absolute position coding, absolute encoder, Gray code, single-track Gray code, necklace

## Abstract

Single-track Gray codes (STGCs) is a type of absolute position coding method for novel angular sensors, because it has single-track property over traditional Gray codes and mono-difference over linear feedback shift register codes. However, given that the coding theory of STGCs is incomplete, STGC construction is still a challenging task even though it has been defined for more than 20 years. Published coding theories and results on STGCs are about two types of STGC, namely, necklace and self-dual necklace ordering, which are collectively called as *k*-spaced head STGCs. To find a new code, three constraints on generating sequences are proposed to accelerate the searching algorithm, and the complete searching result of length-6 STGCs is initially obtained. Among the entire 132 length-6 STGCs, two novel types of STGCs with non-*k*-spaced heads are found, and the basic structures of these codes with the general length *n* are proposed and defined as twin-necklace and triplet-necklace ordering STGCs. Furthermore, *d*-plet-necklace ordering STGC, which unifies all the known STGCs by changing the value of *d*, is also defined. Finally, a single-track absolute encoder prototype is designed to prove that STGCs are as convenient as the traditional position coding methods.

## 1. Introduction

**Figure 1:**
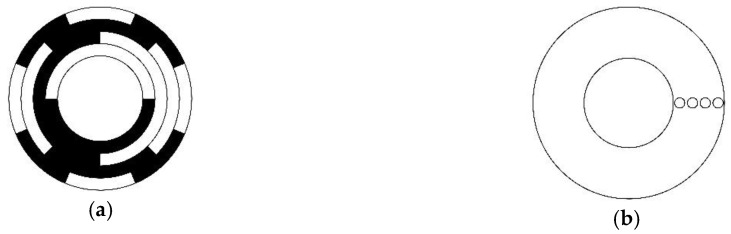
Disc pattern and reading head distribution of absolute encoder using a length-4 Gray code: (**a**) Schematic of the coding disc, where the white area indicates “0”, and the black area indicates ”1”; (**b**) Schematic of the reading disc, where the four small circles denote the four reading heads corresponding to the four coding tracks.

Absolute angular measurement is the critical part for the accuracy of many precision control systems, such as the positioning of aerospace cameras, star positioning in astronomical observation, and precision weapon positioning in the national defense area [1,2,3,4,5]. The most widely used angular sensor is the absolute rotary encoder in which the coding disc carries all the unique angular information for each position in a magnetic or an optical way that can be recovered immediately after a power outage [6,7]. The majority of the existing coding discs are encoded by reflected Gray codes [8,9,10,11], which were originally designed to prevent spurious output from the pulse code transmission because any two adjacent codewords differ in exactly one bit. This unique property of Gray code, which enhances the reliability of absolute position coding, is called mono-difference in this study. A coding disc pattern of length-4 optical absolute encoder is shown in Figure 1, where every codeword etched in radial direction uniquely identifies one angular position in the revolution, and the mono-difference prevents the quantization errors caused by the inconsistent performance of the reading heads. Therefore, 16 different positions can be distinguished by a length-4 reflected Gray code. However, every concentric track on the coding disc denotes one bit of Gray code; thus, the only drawback of any devices making use of such code is the increasing in diameter required by high resolution. 

One widely accepted solution to this drawback is the linear feedback shift register (LFSR) sequence [12], which is also called maximal-length sequence, because its codewords are arranged along the circumferential direction, such that all the position information can be encoded within only one track [13,14]. Unfortunately, LFSR sequences do not generally possess the mono-difference required for error resilience as Gray codes do; thus, the additional synchronization track and highly complicated reading system are inevitable for any devices that use these sequences [15].

**Figure 2:**
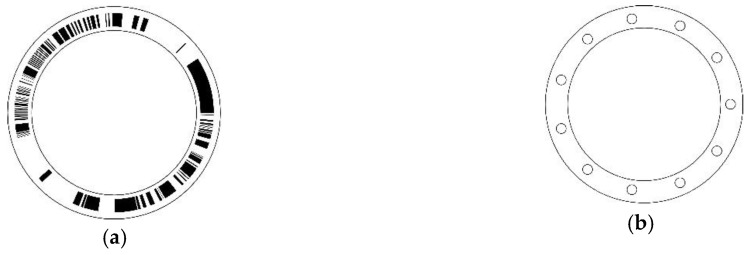
Disc pattern and reading head distribution of absolute encoder using a length-11 period-2046 STGC. (**a**) Schematic of the coding disc, where the white area indicates “0”, and the black area indicates “1”; (**b**) Schematic of the reading disc, where the 11 small circles denote the 11 reading heads and are evenly distributed around the coding track.

Suitable coding methods for absolute angular position which satisfy mono-difference with few code tracks had been studied for years until two patents presented some coding schemes where only one track is adequate to encode all codeword information [16,17]. In 1996, Hiltgen, Paterson, and Brandestini formally defined this type of code as single-track Gray code (STGC) where the single-track property is added to the Gray code, such that every column of the codeword matrix is a cyclic shift of the first one [18]. In the field of absolute position sensing and angular measurement, STGC has “single-track” advantage over conventional Gray codes and “mono-difference” advantage over LFSR sequences, such that it becomes a suitable alternative to other coding methods. Moreover, STGCs reach the limitation of the miniaturization for encoders in the aspect of the absolute position coding theory, where only one coding track is adequate for any resolution [19,20]. One disc pattern encoded by a length-11 period-2046 STGC is shown in Figure 2, where 11 reading heads are uniformly distributed around the single track on the reading disc. Two types of STGC, necklace and self-dual necklace ordering, have been intensively studied [21]. Two iterative constructions for these types were presented in [22]. A survey of the main results on these two types of STGC can be found in [23].

STGC construction remains a challenge although it has been defined for more than 20 years [24]. We only know two structures of STGCs, namely, necklace and self-dual necklace ordering, which are collectively known as *k*-spaced head STGCs. The existing problem of the non-*k*-spaced head STGCs has been proposed as an interesting research topic in a survey [23], which is still unsolved. In the present study, we prove the existence of non-*k*-spaced head STGCs using two new types of code found in the complete searching of length-6 STGCs. On the basis of these codes, two new structures are proposed for length-*n* STGCs, which are defined as twin-necklace and triplet-necklace ordering. The structure of the *d*-plet-necklace ordering for length-*n* STGCs, which unifies all the known types of STGC, is also presented in the present work. Finally, an absolute encoder prototype is proposed using STGCs to promote the use of this code.

## 2. STGCs with *k*-Spaced Heads

**Definition** **1.** *[STGC]: A length n period P binary code:*

$$\left[\begin{array}{cccc} w_{0}^{0}, & w_{0}^{1}, & \cdots , & w_{0}^{n-1}\\ w_{1}^{0}, & w_{1}^{1}, & \cdots , & w_{1}^{n-1}\\ & & \vdots & \\ w_{i}^{0}, & w_{i}^{1}, & \cdots , & w_{i}^{n-1}\\ & & \vdots & \\ w_{P-1}^{0}, & w_{P-1}^{1}, & \cdots , & w_{P-1}^{n-1} \end{array}\right]$$

*is an STGC if and only if it fulfills the following three properties:*

*(1)*  *Single-track property: Each column $W^{j}$ is the cyclic shift of the first one $W^{0}$, i.e., $W^{j}=E^{k_{j}}W^{0}$, where 0≤j<n and $E^{k_{j}}$ denotes $k_{j}$ times cyclic shifting, where $k_{0}=0$.*
*(2)*  *Mono-difference: Any two adjacent codewords, denoted as $W_{i}$ and $W_{i+1}$, differ in only one bit which holds for the first and the last codewords as well.*
*(3)*  *Distinctness: Every codeword is distinct from others.*

**Definition** **2.** *[head position, head interval]: In order to represent all the equivalent STGCs obtained by rearranging the columns, we define head position of a STGC, H_P_, as the ascending order of $\left[k_{0},k_{1},\cdots ,k_{n-1}\right]$:*

$$H_{P}=\mathrm{ascending}\_\mathrm{order}([k_{0},k_{1},\cdots ,k_{n-1}])=[p_{0},p_{1},\cdots ,p_{n-1}],$$

*and the sequence composed of the differences of every adjacent elements in head position is defined as head interval, H_I_:*

$$H_{I}=[p_{1}-p_{0},p_{2}-p_{1},\cdots ,p_{0}-p_{n-1}]=[h_{0},h_{1},\cdots ,h_{n-1}],$$

*where $p_{0}-p_{n-1}$ is reduced modulo P.*

**Definition** **3.** *[section, generating sequence]: We defined a section as a maximal subsequence of consecutive “1” s or consecutive “0” s. Then we define a generating sequence of $W^{0}$ as a sequence of lengths of consecutive sections, assuming that $W^{0}$ starts with a section of “1”s.*

Therefore, a STGC can be briefly represented by generating sequence and head interval. For example, a length-6 period-12 STGC is shown as follows:

$${\left[\begin{array}{l} 110001111111\\ 000111111111\\ 111111000111\\ 111100011111\\ 011111111100\\ 111111110001 \end{array}\right]}^{T}.$$

The cyclic shifting time for each column is $k_{0}=0,k_{1}=2,k_{2}=8,k_{3}=10,k_{4}=4,k_{5}=6$, and according to Definition 2 the head position is $H_{P}=[0,2,4,6,8,10]$, so the head interval is $H_{P}=[2,2,2,2,2,2]$. A brief description of this STGC is the generating sequence [9, 3] and the head interval [2, 2, 2, 2, 2, 2].

Necklace and self-dual necklace ordering STGCs are the only two known types of STGC since it was defined by Etzion and Paterson in 1996, whose construction was based on necklace and self-dual necklace sets in combinatorial theory. These two types of STGC have their reading heads evenly distributed. They are also called *k*-spaced heads STGCs.

### 2.1. Necklace Ordering STGCs

**Definition** **4.** *[necklace]: Let a length n codeword be $W=w_{0}w_{1}\cdots w_{n-1},$ and the cyclic shift of W can be represented as, $E^{l}W=w_{l}w_{l+1}\cdots w_{n-1}w_{0}w_{1}\cdots w_{l-1}$, where **E** is a cyclic shift operator. The cyclic order of a length n codeword W is defined as the minimum period under cyclic shift, i.e., so a necklace is defined as the codeword set:*

$$\left\{W,E^{1}W,E^{2}W,\cdots ,E^{o(W)-1}W\right\},$$

*if o(W)=n, the set is called full-order necklace, otherwise, it is called non-full-order necklace [25,26,27].*

For a length-6 codeword 010001, the necklace set is {010001, 100010, 000101, 001010, 010100, 101000}. A non-full-order necklace If the number of the elements in one necklace set equals the length of its codeword, then the necklace set {001001, 010010, 100100} can be obtained from codeword 001001.

**Theorem** **1.** *[necklace ordering STGC]: Let $S_{0},S_{1},\cdots ,S_{r-1}$ be the seed codes which are from r different full-order length n necklaces, and any two adjacent seed codes differ in exactly one bit, including $S_{r-1}$ and $E^{l}S_{0}$ with an integer l relatively prime to n, then the following codeword list form a length n period P=nr STGC:*

$$\begin{array}{cccc} S_{0}, & S_{1}, & \cdots , & S_{r-1},\\ E^{l}S_{0}, & E^{l}S_{1}, & \cdots , & E^{l}S_{r-1},\\ E^{2l}S_{0}, & E^{2l}S_{1}, & \cdots , & E^{2l}S_{r-1},\\ \vdots & \vdots & \vdots & \vdots \\ E^{(n-1)l}S_{0}, & E^{(n-1)l}S_{1}, & \cdots , & E^{(n-1)l}S_{r-1}, \end{array}$$

*which is called necklace ordering STGC.*

For example, two codes, 100111 and 101111, are selected as the seed codes according to the two conditions above, and a length-6 period-12 necklace ordering STGC can be constructed when the codewords of the two necklace sets are alternatively arranged as follows:

$${\left[\begin{array}{l} 110001111111\\ 000111111111\\ 011111111100\\ 111111110001\\ 111111000111\\ 111100011111 \end{array}\right]}^{T}.$$

Therefore, the appropriate seed codes must be found to construct necklace ordering STGCs; for a short length, a Karnaugh map is convenient for selecting the seed codes [28], whereas a computer searching program is needed for lengths that are higher than 12. 

The brief description of this STGC is also with the generating sequence [9, 3] and the head interval [2, 2, 2, 2, 2, 2], so these two STGCs are the same.

**Figure 3:**
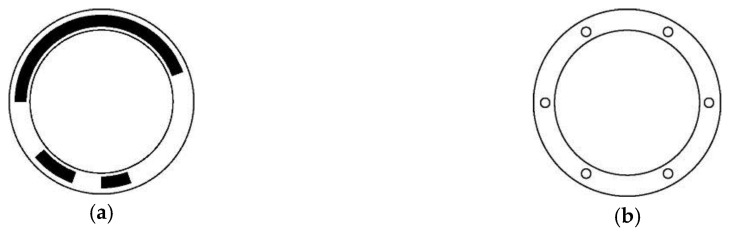
Disc pattern and reading head distribution of absolute encoder using a length-6 period-36 necklace ordering STGC. (**a**) Schematic of the coding disc, where white the area indicates “0”, and the black area indicates “1”; (**b**) Schematic of the reading disc, where the six small circles denote the six reading heads and are evenly distributed around the whole coding track.

The structure is based on necklace sets; thus, the head interval of necklace ordering STGCs is consistently perfectly uniform. Figure 3 shows the coding pattern and reading head distribution of a length-6 period-36 necklace ordering STGC with generating sequence [16, 9, 2, 2, 3, 4] and head interval [6, 6, 6, 6, 6, 6].

### 2.2. Self-Dual Necklace Ordering STGCs

A self-dual word *W* is a word for which its complement is the cyclic shift of *W*. So let $W=S\cdot \bar{S}$ be a length 2*n* self-dual word, where $\bar{S}$ denotes the length *n* word obtained by complementing every bit of *S.* The complementary cyclic shift operator $\bar{E}$ is defined as $\bar{E}S=s_{1},s_{2},\cdots ,s_{n-1},{\bar{s}}_{0},$ where ${\bar{s}}_{0}$ is the binary complement of $s_{0}.$ The *complementary cyclic order* of a length *n* codeword *S* is defined as $\bar{o}(S)=\min\left\{i|{\bar{E}}^{i}S=S,1\leq i\leq 2n\right\}.$

It is obvious that a length 2*n* self-dual codeword is only determined by the length *n* word S, called *base word*. The self-dual necklace can be defined as follows.

**Definition** **5.** *[self-dual necklace]: Let a length 2n self-dual codeword be $W=S\cdot \bar{S}=s_{0},\cdots ,s_{n-1},{\bar{s}}_{0},\cdots ,{\bar{s}}_{n-1}$. A self-dual necklace is defined as the codeword set $\left\{W,EW,E^{2}W,\cdots ,E^{o(W)-1}W\right\}$, and for simplicity a base word set $\left\{S,\bar{E}S,{\bar{E}}^{2}S,\cdots ,{\bar{E}}^{\bar{o}(S)-1}S\right\}$ is also used as the representative of a self-dual necklace. If o(W)=2n or $\bar{o}(S)=2n$, the set is called full-order self-dual necklace, otherwise, it is called non-full-order self-dual necklace [29,30,31].*

Another classic type of STGC is the self-dual necklace ordering STGCs, which has a structure similar to necklace ordering STGCs.

**Theorem** **2.** *[self-dual necklace ordering STGCs]: Let $S_{0},S_{1},\cdots ,S_{r-1}$ be the seed code, which consists of r base words from r different full-order length 2n self-dual necklaces, and any two adjacent seed codes differ only in one bit which also holds for $S_{r-1}$ and ${\bar{E}}^{l}S_{0}$ with an integer l relatively prime to 2n, then the following word list form a length n period P=2nr single-track Gray code:*

$$\begin{array}{cccc} S_{0}, & S_{1}, & \cdots , & S_{r-1},\\ {\bar{E}}^{l}S_{0}, & {\bar{E}}^{l}S_{1}, & \cdots , & {\bar{E}}^{l}S_{r-1},\\ {\bar{E}}^{2l}S_{0}, & {\bar{E}}^{2l}S_{1}, & \cdots , & {\bar{E}}^{2l}S_{r-1},\\ \vdots & \vdots & \vdots & \vdots \\ {\bar{E}}^{(n-1)l}S_{0}, & {\bar{E}}^{(n-1)l}S_{1}, & \cdots , & {\bar{E}}^{(n-1)l}S_{r-1},\\ \vdots & \vdots & \vdots & \vdots \\ {\bar{E}}^{(2n-1)l}S_{0}, & {\bar{E}}^{(2n-1)l}S_{1}, & \cdots , & {\bar{E}}^{(2n-1)l}S_{r-1}, \end{array}$$

*which is called self-dual necklace ordering STGC.*

**Figure 4:**
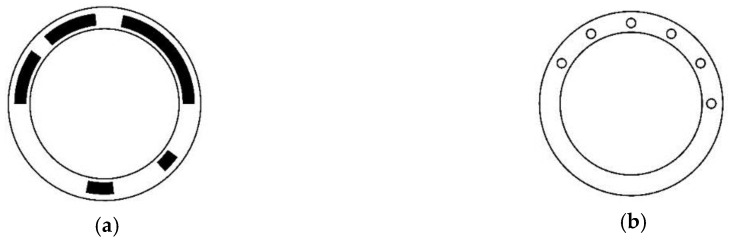
Disc pattern and reading head distribution of absolute encoder using a length-6 period-36 necklace ordering STGC. (**a**) Schematic of the coding disc, where the white area indicates “0”, and the black area indicates “1; (**b**) Schematic of the reading disc, where the six small circles denote the six reading heads and are evenly distributed around the half coding track.

The only difference of self-dual necklace ordering STGCs from necklace ordering STGCs is the seed codes should be self-dual words. Since the last *n* bits of a length 2*n* self-dual word are redundant, self-dual necklace ordering STGCs are the special case of necklace ordering after deleting these redundant information. Therefore, the reading heads are distributed along the half of the coding disc (the reading heads on the other half are deleted), and the interval is also evenly distributed. Figure 4 shows the disc pattern and reading head distribution of a length-6 period-60 self-dual necklace ordering STGC with generating sequence [13, 3, 6, 2, 6, 13, 3, 6, 2, 6] and head interval [5, 5, 5, 5, 35].

The generating sequence of a self-dual necklace ordering STGC is also self-dual; thus, each reading head has two positions where it can be arranged, which are 180° apart. Therefore, five new head intervals [5, 5, 5, 10, 25], [5, 5, 10, 5, 20, 15], [5, 5, 15, 20, 5, 10], [5, 10, 10, 15, 10, 10], and [5, 15, 5, 15, 5, 15], which can also construct STGCs with the same generating sequence shown in Figure 4, are available. These transformed STGCs from self-dual necklace ordering are the only published codes with non-*k*-spaced heads.

## 3. STGCs with Non-*k*-Spaced Head

### 3.1. Complete Solution of Length-6 STGCs

Necklace and self-dual necklace ordering are the only known structures of STGCs. The main open problem for STGCs was proposed by Etzion regarding the presence of STGCs with non-*k*-spaced heads except the transformed codes from self-dual necklace orderings [21,22,23]. A computer program was used in searching the complete solution of length-*n* STGCs based only on the definition of STGCs to find new words.

STGCs are rare among binary codes. For length-5 period-30 codes, $2^{30}$ sequences can possibly be the generating sequences; however, only 13 sequences generate STGCs. In view of complex computation time, Yan and Wang proposed a speed-up algorithm to obtain the complete solution for length-5 STGCs [32]. However, this algorithm is insufficient for length-6 codes. Thus, we propose three constraints to identify the impossible generating sequences rapidly and finally find the complete solution for length-6 STGCs.

#### 3.1.1. Constraints for the Generating Sequence of STGCs

● Constraint 1: Number of the sections of the generating sequence:

For length-*n* period-*P* STGCs, we assume the presence of *m* sections in the generating sequence; thus, *m* junctions of two adjacent sections exist. Each junction causes mono-difference of two adjacent words, and a total of n×m junctions exists in the codeword matrix. Since *P* words must have mono-difference, n×m junctions equal to *P*; thus, the number of sections *m* must be *P*/*n*. Moreover, if the beginning and ending sections consist of the same component, then they are regarded as one section; thus, *the number of the sections of the generating sequence, m, must be even*. 

● Constraint 2: Minimum section length of the generating sequence:

*For length-n period-P STGCs, the minimum section length of its generating sequence is at least 2*. Having a section length less than 2 and, subsequently, two equal codewords in the matrix is a contradiction of distinctness.

● Constraint 3: Maximum section length of the generating sequence:

*For length-n period-P STGCs, the maximum section length of its generating sequence is at least n*. *P*/*n* sections exist in the generating sequence; thus, if the maximum section length is less than *n*, then the length of the generating sequence must be less than *P*, which is impossible.

#### 3.1.2. Result of Length-6 STGCs

A month is required to complete the searching of the 132 length-6 STGCs using a personal computer, where some new non-*k*-spaced head codes are found, as shown in Table 1. The complete results of length-6 STGCs can be found in Appendix B.

### 3.2. Twin-Necklace Ordering STGCs

The head intervals of the new STGCs are periodically circulating, and some of the period is 2. We define these codes as twin-necklace ordering STGCs, and the structure of these new codes will be introduced.

**Definition** **6.** *[twin-necklace]: Let a length n codeword W be a combination of two length n/2 sub-words, $W=W_{1}\cdot W_{2}$. A twin-necklace set consists of the following codewords*

$$\left\{W_{1}\cdot W_{2},EW_{1}\cdot EW_{2},E^{2}W_{1}\cdot E^{2}W_{2},\cdots ,E^{\mathrm{lcm}\left[o(W_{1}),o(W_{2})\right]}W_{1}\cdot E^{\mathrm{lcm}\left[o(W_{1}),o(W_{2})\right]}W_{2}\right\},$$

*where l cm denotes the least common multiple and $o(W_{1})=\min\left\{i|E^{i}W_{1}=W_{1},1\leq i\leq n/2\right\}$. If the number of the elements in one length n twin-necklace set is n/2, the set is called full-order twin-necklace.*

For example, the twin-necklace set of a length-6 word 011001 is {011001, 110010, 101100}, which is obtained by cyclic shifting the first half word 011 and the second half 001 independently.

**Theorem** **3.** *[twin-necklace ordering STGCs]: Let $S_{0}\cdot S_{t},S_{1}\cdot S_{t+1},\cdots ,S_{r-t}\cdot E^{l}S_{0},\cdots ,S_{r-1}\cdot E^{l}S_{t-1}$ be the r seed codes which are from r different length n full-order twin-necklaces. The last n/2 columns of the seed codes ${\left[S_{t},S_{t+1},\cdots ,S_{r-1},E^{l}S_{0},\cdots ,E^{l}S_{t-1}\right]}^{T}$ is the t time cyclic shift of the first n/2 columns ${\left[S_{0},S_{1},\cdots ,S_{r-1}\right]}^{T}$, where 1≤t≤r/2−1. Any two adjacent seed codes differ only in one bit which also holds for $S_{r-1}\cdot E^{l}S_{t-1}$ and $E^{l}S_{0}\cdot E^{l}S_{t}$ with l relatively prime to n/2, then the word list:*

$$\begin{array}{cccccc} S_{0}\cdot S_{t}, & S_{1}\cdot S_{t+1}, & \cdots , & S_{r-t}\cdot E^{l}S_{0}, & \cdots , & S_{r-1}\cdot E^{l}S_{t-1},\\ E^{l}S_{0}\cdot E^{l}S_{t}, & E^{l}S_{1}\cdot E^{l}S_{t+1}, & \cdots , & E^{l}S_{r-t}\cdot E^{2l}S_{0}, & \cdots , & E^{l}S_{r-1}\cdot E^{2l}S_{t-1},\\ \vdots & \vdots & \vdots & \vdots & \vdots & \vdots \\ E^{(n/2-1)l}S_{0}\cdot E^{(n/2-1)l}S_{t}, & E^{(n/2-1)l}S_{1}\cdot E^{(n/2-1)l}S_{t+1}, & \cdots , & E^{(n/2-1)l}S_{r-t}\cdot S_{0}, & \cdots , & E^{(n/2-1)l}S_{r-1}\cdot S_{t-1}, \end{array}$$

*forms a length n period P=nr/2 STGC which is called a twin-necklace ordering STGC.*

The proof of Theorem 3 can be found in Appendix A.

A computer program is needed to find the suitable seed codes that satisfy the properties in Theorem 3. For example, for constructing a length-6 period-24 twin-necklace ordering STGC, we select eight length-3 sub-words 001, 001, 000, 000, 001, 001, 011, 011 as the first three columns of the seed codes. We choose t=3 and l=1, so the seed codes are:

$${\left[\begin{array}{l} 00000000\\ 00000011\\ 11001111\\ 00000000\\ 00011110\\ 01111000 \end{array}\right]}^{T},$$

which are from eight different twin-necklace sets and satisfy the mono-difference, so using the structure in Theorem 4 to construct all the codewords, a twin-necklace ordering STGC can be obtained. The codeword matrix is shown as follows:

$${\left[\begin{array}{l} 000000000000001111001111\\ 000000111100111100000000\\ 110011110000000000000011\\ 000000000001111001111000\\ 000111100111100000000000\\ 011110000000000000011110 \end{array}\right]}^{T},$$

where the generating sequence is [14, 4, 2, 4] and the cyclic shifting times are $k_{0}=0,k_{1}=8,k_{2}=16,k_{3}=3,k_{4}=11,k_{5}=19$, so the head position is [0,3,8,11,16,19], and the head interval is [3, 5, 3, 5, 3, 5].

The head interval of twin-necklace ordering STGCs is different from any known word. The structure of a length-*n* twin-necklace ordering STGC can be regarded as a combination of two length-*n*/2 necklace ordering STGCs. The first length-*n*/2 necklace ordering leads to *n*/2 reading heads that are uniformly distributed along the entire encoding disc, and the second length-*n*/2 necklace ordering leads to another uniformly distributed *n*/2 reading heads. The second necklace ordering is the shifted equivalent by *t* of the first one; thus, the two groups of *n*/2 reading heads are distributed as follows:

$$\left[t,\frac{2P}{n}-t,t,\frac{2P}{n}-t,\cdots ,t,\frac{2P}{n}-t,\right],$$

where the sub-cycle of the head interval is 2.

**Figure 5:**
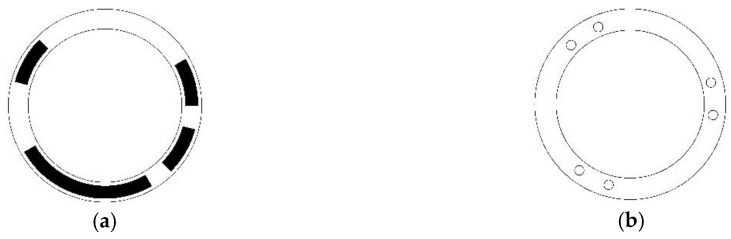
Disc pattern and reading head distribution of absolute encoder using a length-6 period-48 twin-necklace ordering STGC: (**a**) Schematic of the coding disc, where white area indicates “0”, and the black area indicates “1”; (**b**) Schematic of the reading disc, where the six small circles denote the six reading heads, and the sub-cycle of the head interval is two.

Twin-necklace orderings are the first non-*k*-spaced head STGCs, where the periodic head interval is caused by the special structure itself. For example, Figure 5 shows the coding disc and reading head distribution of a length-6 period-48 twin-necklace ordering STGC, where t=3, the generating sequence is [14, 4, 6, 12, 2, 4, 2, 4], and the head interval is [3, 13, 3, 13, 3, 13]. 

The maximum periods of necklace, self-dual necklace and twin-necklace ordering STGCs from length 6 to length 16 are shown in Table 2. The upper bound of period for twin-necklace orderings is not less than necklace orderings and not greater than self-dual necklace orderings:

$$P_{\max\_\mathrm{nekclace}}\leq P_{\max\_\mathrm{twin}}\leq P_{\max\_\mathrm{self}-\mathrm{dual}},$$

and the difference between $P_{\max\_\mathrm{twin}}$ and $P_{\max\_\mathrm{self}-\mathrm{dual}}$ is obvious when *n*/2 can be divisible by more factors.

### 3.3. Triplet-Necklace Ordering STGCs

Another new type of STGC with periodic head interval is found in the complete result of length-6 STGCs with sub-cycle of 3, which are listed in Appendix B Table A4; it is defined as *triplet-necklace ordering STGCs* because it consists of three length-*n*/3 necklace ordering.

**Definition** **7.** *[triplet-necklace]: Let a length n codeword W be a combination of three length n/3 sub-words, $W=W_{1}\cdot W_{2}\cdot W_{3}$. A triplet-necklace set consists of the following codewords:*

$$\left\{W_{1}\cdot W_{2}\cdot W_{3},EW_{1}\cdot EW_{2}\cdot EW_{3},\cdots ,E^{o_{3}(W)}W_{1}\cdot E^{o_{3}(W)}W_{2}\cdot E^{o_{3}(W)}W_{3}\right\}$$

*where $o_{3}(W)=\mathrm{lcm}[o(W_{1}),o(W_{2}),o(W_{3})]$, $o(W_{1})=\min\left\{i|E^{i}W_{1}=W_{1},1\leq i\leq n/2\right\}$ and l cm denotes the least common multiple. If the number of the elements in one length n triplet-necklace set is n/3, the set is called full-order triplet-necklace.*

For example, the triplet-necklace set of a length-6 word 011001 is {011001, 100110}, which is obtained by cyclic shifting the three sub-words 01, 10, and 01 independently.

**Theorem** **4.** *[triplet-necklace ordering STGCs]: Let*

$$\begin{array}{l} S_{0}\cdot S_{t_{1}}\cdot S_{t_{2}},S_{1}\cdot S_{t_{1}+1}\cdot S_{t_{2}+1},\cdots ,S_{r-t_{2}}\cdot S_{t_{1}+r-t_{2}}\cdot E^{l}S_{0},\\ \cdots ,S_{r-t_{1}}\cdot E^{l}S_{0}\cdot E^{l}S_{t_{2}-t_{1}},\cdots ,S_{r-1}\cdot E^{l}S_{t_{1}-1}\cdot E^{l}S_{t_{2}-1} \end{array}$$

*be the r seed codes which are from r different length n full-order triplet-necklace sets. The second n/3 columns of the seed codes $S_{t_{1}},S_{t_{1}+1},\cdots ,S_{r-1},E^{l}S_{0},\cdots ,E^{l}S_{t_{1}-1}$ is the t_1_ time cyclic shift of the first n/3 columns $S_{0},S_{1},\cdots ,S_{r-1}$, and the third n/3 columns $S_{t_{2}},S_{t_{2}+1},\cdots ,S_{r-1},E^{l}S_{0},\cdots ,E^{l}S_{t_{2}-1}$ is the t_2_ time cyclic shift of the first n/3 columns, where $1\leq t_{1}<t_{2}\leq r/3-1$. And any two adjacent seed codes differ only in one bit which also holds for $S_{r-1}\cdot E^{l}S_{t_{1}-1}\cdot E^{l}S_{t_{2}-1}$ and $E^{l}S_{0}\cdot E^{l}S_{t_{1}}\cdot E^{l}S_{t_{2}}$ with l relatively prime to n/3, then the word list:*

$$\begin{array}{cccc} S_{0}\cdot S_{t_{1}}\cdot S_{t_{2}}, & S_{1}\cdot S_{t_{1}+1}\cdot S_{t_{2}+1}, & \cdots , & S_{r-1}\cdot E^{l}S_{t_{1}-1}\cdot E^{l}S_{t_{2}-1},\\ E^{l}S_{0}\cdot E^{l}S_{t_{1}}\cdot E^{l}S_{t_{2}}, & E^{l}S_{1}\cdot E^{l}S_{t_{1}+1}\cdot E^{l}S_{t_{2}+1}, & \cdots , & E^{l}S_{r-1}\cdot E^{2l}S_{t_{1}-1}\cdot E^{2l}S_{t_{2}-1},\\ \vdots & \vdots & \vdots & \vdots \\ E^{(n/3-1)l}S_{0}\cdot E^{(n/3-1)l}S_{t_{1}}\cdot E^{(n/3-1)l}S_{t_{2}}, & E^{(n/3-1)l}S_{1}\cdot E^{(n/3-1)l}S_{t_{1}+1}\cdot E^{(n/3-1)l}S_{t_{2}+1}, & \cdots , & E^{(n/3-1)l}S_{r-1}\cdot S_{t_{1}-1}\cdot S_{t_{2}-1}, \end{array}$$

*forms a length n period P=nr/3 STGC called triplet-necklace ordering STGC.*

The proof of Theorem 4 is analogous to the proof of Theorem 3, which can be found in Appendix A. A computer program is needed to find the suitable seed codes that satisfy the properties in Theorem 4. For example, for a length-6 period-24 triplet-necklace ordering STGCs, we select 12 length-2 sub-words 10, 10, 10, 11, 11, 11, 10, 10, 10, 00, 00, 00 as the seed codes for the first necklace structure. Thus, the first necklace structure is:

$${\left[\begin{array}{l} 111111111000000111000000\\ 000111000000111111111000 \end{array}\right]}^{T}.$$

The numbers of cyclic shift for the second and third necklace structures are $t_{1}=2$ and $t_{2}=4$. Thus, these two necklace structures are:

$${\left[\begin{array}{l} 111111100000011100000011\\ 011100000011111111100000 \end{array}\right]}^{T}\;\mathrm{and}\;{\left[\begin{array}{l} 111110000001110000001111\\ 110000001111111110000001 \end{array}\right]}^{T}.$$

The combination of the first 12 words is the seed codes of the final triplet-necklace ordering, 101011, 101111, 101110, 111110, 111010, 111000, 101000, 100000, 100001, 000001, 000101, 000111. The final codeword matrix with generating sequence of [9, 6, 3, 6] and head interval of [2, 2, 8, 2, 2, 8] is shown as follows:

$${\left[\begin{array}{l} 111111111000000111000000\\ 000111000000111111111000\\ 111111100000011100000011\\ 011100000011111111100000\\ 111110000001110000001111\\ 110000001111111110000001 \end{array}\right]}^{T}.$$

For triplet-necklace ordering STGCs, the reading heads evenly divide the entire coding circle three times. The intervals between the three necklace ordering structures are $t_{1}$ and $t_{2}$; thus, the head interval of a length-*n* period-*P* triplet-necklace ordering STGC is:

$$\left[t_{1},t_{2}-t_{1},\frac{3P}{n}-t_{2},t_{1},t_{2}-t_{1},\frac{3P}{n}-t_{2},\cdots \right],$$

where the sub-cycle is 3.

**Figure 6:**
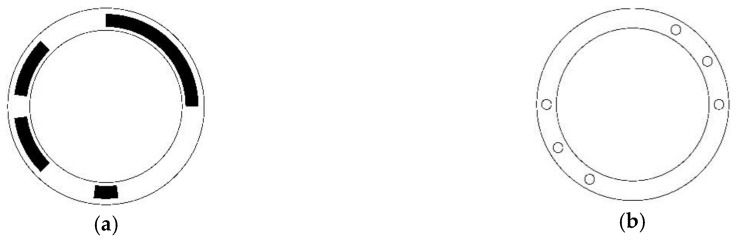
Disc pattern and reading head distribution of absolute encoder using a length-6 period-48 triplet-necklace ordering STGC: (**a**) Schematic of the coding disc, where the white area indicates “0”, and the black area indicates “1”; (**b**) Schematic of the reading disc, where the six small circles denote the six reading heads, and the sub-cycle of the head interval is three.

Figure 6 shows the coding disc and reading head distribution of a length-6 period-48 triplet-necklace ordering STGC, where $t_{1}=4$, $t_{2}=8$, the generating sequence is [12, 6, 5, 2, 5, 5, 2, 11], and the head interval is [4, 4, 16, 4, 4, 16].

### 3.4. D-Plet-Necklace Ordering STGCs

The most intuitive conjecture is whether quadruplet, quintuplet, or even multiple-birth- necklace ordering STGCs exist based on the discovery of twin and triplet-necklace ordering. Indeed, these conjectural multiple-birth-necklace orderings exist. In this part, the *d*-plet-necklace ordering STGC is defined, and some new codes of length-8 and -10 are presented for the first time.

**Definition** **8.** *[d-plet-necklace]: For length n codeword W, if n can be divided by an integer d, then W can be regarded as a concatenation of d length n/d sub-words, $W=W_{0}\cdot W_{1}\cdots W_{d-1}$. Thus, we define a length-n d-plet-necklace set as a combination of d-length n/d necklaces, consisting of the following codewords:*

$$\left\{W_{1}\cdot W_{2}\cdots W_{d-1},EW_{1}\cdot EW_{2}\cdots EW_{d-1},\cdots \cdots ,E^{o_{d}(W)-1}W_{1}\cdot E^{o_{d}(W)-1}W_{2}\cdots E^{o_{d}(W)-1}W_{d-1}\right\},$$

*where $o_{d}(W)=\mathrm{lcm}[o(W_{0}),o(W_{1}),\cdots ,o(W_{d-1})]$, $o(W_{1})=\min\left\{i|E^{i}W_{1}=W_{1},1\leq i\leq n/2\right\}$ and lcm denote the least common multiple. Similarly, if the number of the elements in the set is n/d, then it is called a full-order d-plet-necklace.*

**Theorem** **5.** *[d-plet-necklace ordering STGCs]: Let:*

$$S_{0}\cdot S_{t_{1}}\cdots S_{t_{d-1}},S_{1}\cdot S_{t_{1}+1}\cdots S_{t_{d-1}+1},\cdots \cdots ,S_{r-1}\cdot E^{l}S_{t_{1}-1}\cdots E^{l}S_{t_{d-1}-1}$$

*be the r seed codes which are from r different length n full-order d-plet-necklace sets. The seed codes can be regarded as d groups, and the ith group consists of n/d codewords, $S_{t_{i}},S_{t_{i}+1},\cdots ,S_{r-1},E^{l}S_{0},\cdots ,E^{l}S_{t_{i}-1}$ which is the t_i_ time cyclic shift of the first n/d columns, where $t_{0}=0$ and $1\leq t_{1}<t_{2}<\cdots <t_{d-1}\leq r/d-1$. And any two adjacent seed codes differ only in one bit which also holds for $S_{r-1}\cdot E^{l}S_{t_{1}-1}\cdots E^{l}S_{t_{d-1}-1}$ and $E^{l}S_{0}\cdot E^{l}S_{t_{1}}\cdots E^{l}S_{t_{d-1}}$ with l relatively prime to n/d, then the word list:*

$$\begin{array}{ccc} S_{0}\cdot S_{t_{1}}\cdots S_{t_{d-1}}, & \cdots \cdots , & S_{r-1}\cdot E^{l}S_{t_{1}-1}\cdots E^{l}S_{t_{d-1}-1},\\ E^{l}S_{0}\cdot E^{l}S_{t_{1}}\cdots E^{l}S_{t_{d-1}}, & \cdots \cdots , & E^{l}S_{r-1}\cdot E^{2l}S_{t_{1}-1}\cdots E^{2l}S_{t_{d-1}-1},\\ \vdots & \vdots & \vdots \\ E^{(n/d-1)l}S_{0}\cdot E^{(n/d-1)l}S_{t_{1}}\cdots E^{(n/d-1)l}S_{t_{d-1}}, & \cdots \cdots , & E^{(n/d-1)l}S_{r-1}\cdot S_{t_{1}-1}\cdots S_{t_{d-1}-1}, \end{array}$$

*form a length n period P=nr/d STGC called d-plet-necklace ordering STGC.*

The proof of Theorem 5 is analogous to the proof of Theorem 3, which can be found in Appendix A. The structure of a length-*n d*-plet-necklace ordering STGC is the combination of *d*-length *n*/*d* necklace orderings. The *i*th necklace ordering is the $t_{i}$ time cyclic shift of the first one, where i=1,2,⋯,d−1. So for a *d*-plet-necklace ordering STGC, the reading heads evenly divide the entire coding circle for *d* times. The intervals between the *d*-plet-necklace ordering structures are $t_{1},t_{2},\cdots ,t_{d-1}$; thus, the head interval of a length-*n* period-*P d*-plet-necklace ordering STGC is:

$$\left[t_{1},t_{2}-t_{1},\cdots ,t_{d-1}-t_{d-2},\frac{dP}{n}-t_{d-1},t_{1},t_{2}-t_{1},\cdots ,t_{d-1}-t_{d-2},\frac{dP}{n}-t_{d-1},\cdots \cdots \right],$$

where the sub-cycle of the head interval is *d*.

A computer program is also needed to find the suitable seed codes that satisfy the properties in Theorem 6. Some new STGCs are found by this *d*-plet-necklace ordering structure, and examples of 4-plet- and 5-plet-necklace ordering STGCs are listed in Table 3.

## 4. Reclassification of STGCs

When d=1, this *d*-plet-necklace ordering has the structure of a traditional necklace ordering STGC, and when d=n, it is the traditional self-dual necklace ordering STGC. When d=2 or d=3, the *d*-plet- necklace ordering STGCs are twin- or triplet-necklace ordering. Therefore, *d*-plet-necklace ordering STGCs unify all the known STGCs.

## 5. Absolute Encoder Prototype Using STGCs

The majority of the absolute encoders used in industrial applications are encoded by traditional Gray codes. We design an absolute encoder prototype using STGCs, which has been proven convenient as the traditional Gray codes, to promote the use of STGCs.

### 5.1. Coding and Slit Discs

**Figure 7:**
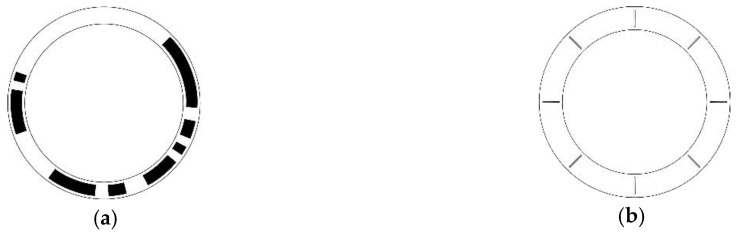
Disc pattern and slit disc of the prototype using a length-8 period-128 STGC: (**a**) Schematic of the coding disc, where the white area indicates “0”, and the black area indicates “1; (**b**) Schematic of the slit disc, where the eight slits are arranged right over the eight reading heads. This disc except the eight slits should be black, but to show the slits clearly we use white instead.

A length-8 period-128 necklace ordering STGC is used for the coding disc with generating sequence of [41, 2, 2, 10, 12, 11, 3, 4, 5, 8, 2, 2, 2, 4, 3, 17], as shown as in Figure 7. A slit disc, where eight slits are uniformly distributed along the circle of which the diameter equals to that of the coding disc, is designed to eliminate light interference. 

The coding disc is attached to the shaft of the encoder by an ultraviolet-ray-stuck adhesive, which is the only part that rotates with the shaft in the prototype. The slit disc, which is a black film disc with eight uniformly distributed narrow white areas that allow the passing of light, is attached to the encoder body right over the eight reading heads with clearance of 1 mm.

**Figure 8:**
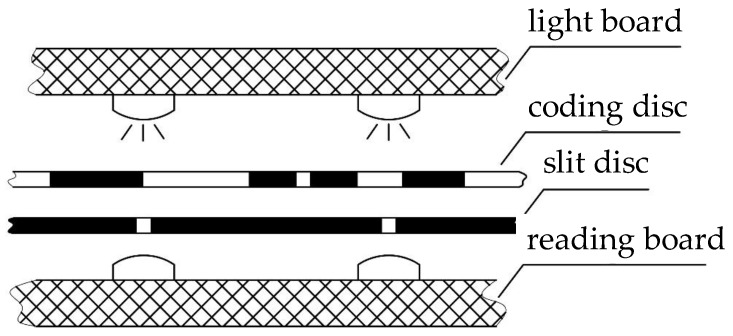
The structural schematic of the prototype.

**Figure 9:**
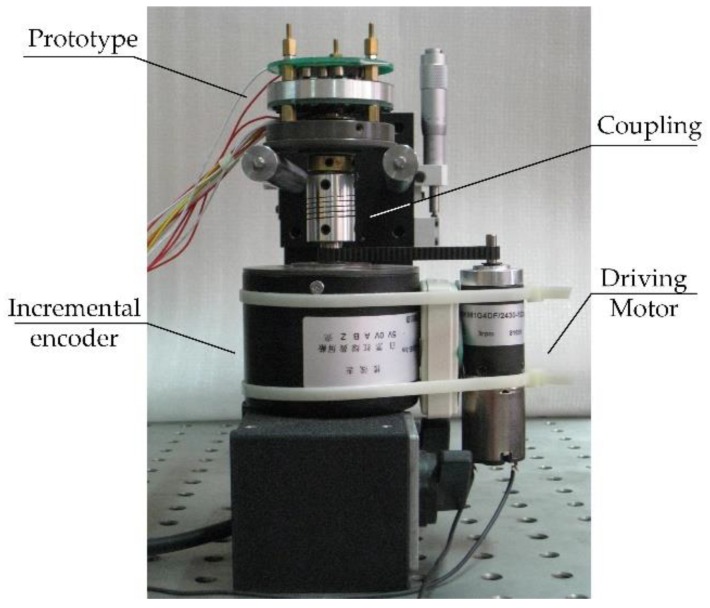
Experimental system.

Eight pairs of OL87KLB infrared light-emitting diodes (LEDs) and OTD10KLCG photo-transistors are employed as the reading heads to recover the information on the coding disc. Eight LEDs are surface mounted on a printed circuit board (PCB) as the top part of the encoder, and eight photo-transistors along with the decoding part are surface mounted on another PCB under the slit disc. The output of the photo-transistors is sent to the decoding memory as an input after shaping by a set of Schmidt triggers. From top to bottom, the PCBs of the LEDs and photo-transistors and the coding and slit discs are laid in the prototype, as shown in Figure 8. A photo of the experimental system is shown in Figure 9.

### 5.2. Decoding

A large memory space rather than decoding circuits is used to decode the codewords of STGCs into traditional Gray codes. An additional function called zero setting makes the operation more convenient in selecting any of the 128 positions as zero position by only pressing a button. This flexible function is urgently desired for various applications to eliminate the inconvenience in locating the zero pulse manually, such as in the traditional position coding methods.

A flash memory, which has 128 times larger storage capacity than the traditional method, is required to store position values that are determined by codeword information and zero position. The address of the flash is the combination of the zero position and real-time codeword. Every time the button is pressed, a new zero position will be locked into a nonvolatile memory FM1110 and sent to be the high 8-bit codeword of the flash address. At the same time, the real-time codeword read by the reading heads is sent to be the low 8-bit of the flash address.

When the low and high 8-bit codewords are identical, the combined 16-bit codeword should be decoded to position “0”. As the shaft rotates forward, the low 8-bit codeword changes to be the consecutive codewords that should be decoded to positions “1”, “2”, “3”, …, “126”, and “127”. All 128 zero positions can be decoded this way, and positions “0”–“127” are decoded into 8-bit reflected Gray codes “00000000”–“01000000” to avoid output coarse error. The unused memory space is filled with codeword “11111111,” which will be used to detect the input mistakes if it is an impossible position value obtained from the flash memory output. All the decoding information is burnt into the flash memory S29GL256N, which is used as a look-up table. 

### 5.3. Error Analysis

The prototype of period-128 single-track absolute encoder is designed with a diameter of 50 mm, and its performance is evaluated by the experiment system shown in Figure 9. An 8192 ppr LFA-500A/501 incremental encoder with error of 0.5′ is used as a benchmark in the experiment system and is driven by a low-speed DC motor together with the prototype.

**Figure 10:**
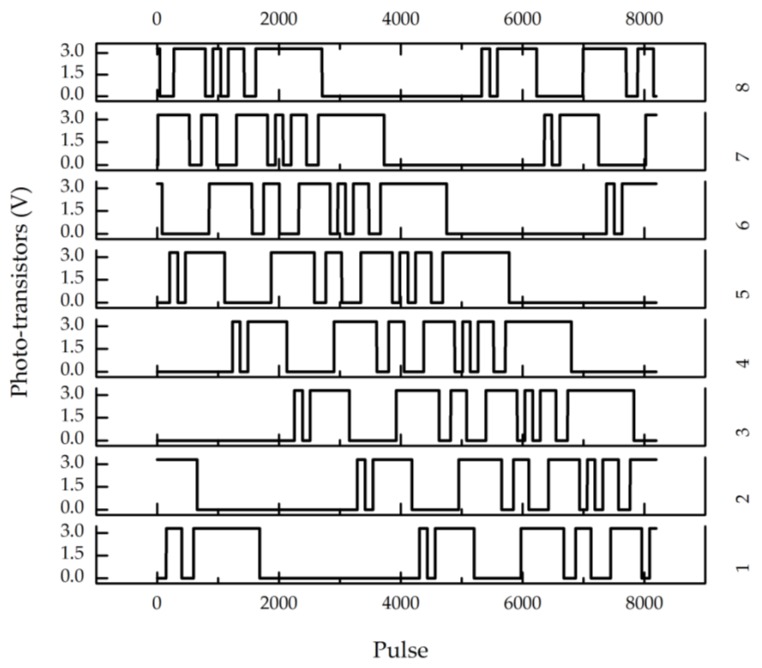
Outputs of the eight reading heads.

The outputs of the eight reading heads are transferred to an acquisition card DAQ-2204 at 3000 Hz sampling rate. The outputs of reading heads 1–8 in a complete revolution are shown in Figure 10, where the abscissa denotes the pulses of the incremental encoder, which are 8192 pulses per revolution and 64 pulses for every position. 

Every channel has 16 sections with maximum relative error of 0.73% caused by transistor 7 when every section length is recovered. In comparison with the theoretical phase difference between two adjacent channels (1024 pulses), a maximum relative error of 0.96% occurred between transistors 6 and 7.

The final output of the prototype is converted to Binary-Coded Decimal (BCD) code by a display module to show the real-time position value. After the power has been switched on, the shaft of the prototype rotates at a speed of 1 rpm; meanwhile, the LEDs on the display module show the corresponding position values. Then, once the zero-setting button is pressed, the LEDs will immediately show the position values with respect to the new zero position.

**Figure 11:**
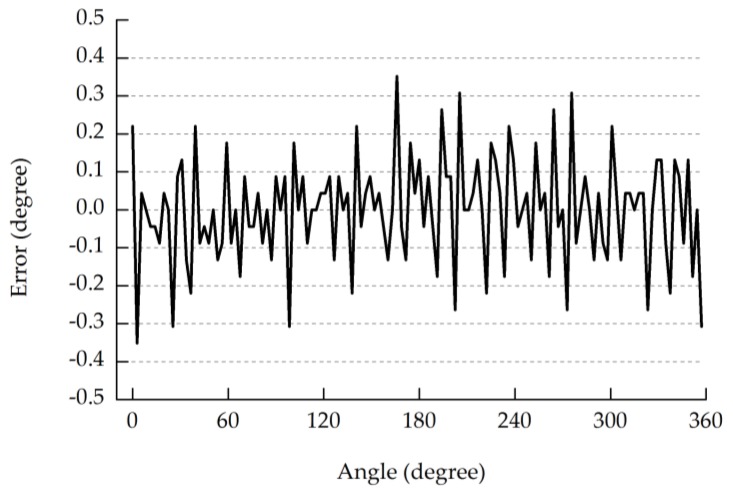
Error of the outputs of the prototype.

As the shaft rotates, the outputs of the prototype and the incremental encoder are acquired by DAQ-2204. The error of a complete rotation is shown in Figure 11. In a complete revolution, the maximum error is 0.35°, which is equivalent to 1/8 of its resolution (360°/128 = 2.82°).

The error shown in Figure 11 is a combination error, which is only affected by the optical, mechanical, and electrical manufacturing processes. Therefore, no matter what type of STGCs is used, the error analysis result will be the same under the same manufacturing and experiment conditions. 

## 6. Discussion and Conclusions

The complete searching result of length-6 STGCs is obtained for the first time. Based on these new codes, twin-necklace ordering, triplet-necklace ordering and *d*-plet necklace ordering STGCs are defined, which are STGCs with non-*k*-spaced heads and the periodic head intervals are with sub-cycle lengths of 2, 3 and *d*, respectively. Moreover, the basic structures of these new STGCs are proposed for the general length *n*, and *d*-plet necklace ordering STGC unifies all the known subclasses of STGCs.

These new STGCs with non-k-spaced heads improve the previous theory and results of STGCs, and the prototype have proved that STGCs can easily replace the traditional Gray codes in practical applications. However, since non-full-order sets exist in all kinds of necklace sets, not all the length *n* binary codes can be the codewords of a length *n* STGC. Therefore, the maximum period of length-*n* STGCs is $P_{\max}<2^{n}$, and the upper bound on periods of all types STGCs of length-*n* is the important problem that should be solved from the point of view of further applications.

## Figures and Tables

**Table 1:** New STGCs with non-*k*-spaced heads in the complete searching result of length-6 codes.

Period	Generating Sequence	Head Interval	Sub-Cycle of Head Interval
12	[6, 6]	[1, 3, 1, 3, 1, 3]	2
24	[14, 4, 2, 4]	[3, 5, 3, 5, 3, 5]	2
	[9, 6, 3, 6]	[2, 2, 8, 2, 2, 8]	3
36	[14, 5, 6, 5, 2, 4]	[3, 9, 3, 9, 3, 9]	2
	[11, 2, 5, 11, 2, 5]	[3, 9, 3, 9, 3, 9]	2
48	[15, 8, 6, 5, 3, 3, 2, 6]	[4, 12, 4, 12, 4, 12]	2
	[14, 6, 2, 4, 2, 6, 6, 8]	[3, 13, 3, 13, 3,13]	2
	[12, 6, 5, 2, 5, 5, 2, 11]	[4, 4, 16, 4, 4, 16]	3
60	[12, 2, 4, 6, 4, 8, 6, 4, 4, 10]	[9, 11, 9, 11, 9, 11]	2

**Table 2:** The upper bounds of period for necklace, self-dual necklace and twin-necklace ordering STGCs from length 6 to length 16.

*n*	$P_{max\_necklace}$	$P_{max\_self-dual}$	$P_{max\_twin}$
6	48	60	60
8	224	240	240
10	960	1020	1020
12	3960	4056	3960
14	16128	16380	16380
16	65024	65504	65280

**Table 3:** Examples of *d*-plet-necklace ordering STGCs.

*d*	*n*	Period	Generating Sequence	Head Interval
4	8	80	[20, 7, 6, 6, 2, 6, 6, 6, 2, 19]	[5, 5, 5, 25, 5, 5, 5, 25]
4	8	96	[15, 8, 3, 13, 8, 3, 5, 15, 2, 7, 15, 2]	[6, 6, 6, 30, 6, 6, 6, 30]
4	8	112	[16, 16, 3, 8, 5, 11, 5, 3, 8, 5, 3, 13, 11, 5]	[6, 6, 6, 38, 6, 6, 6, 38]
4	8	128	[22, 4, 18, 10, 4, 18, 9, 2, 2, 2, 5, 21, 2, 2, 2, 5]	[8, 8, 8, 40, 8, 8, 8, 40]
5	10	120	[35, 22, 13, 5, 2, 3, 10, 7, 3, 10, 5, 5]	[4, 4, 4, 4, 44, 4, 4, 4, 4, 44]
5	10	140	[25, 25, 10, 15, 5, 5, 5, 10, 5, 5, 5, 10, 10, 5]	[4, 4, 4, 4, 54, 4, 4, 4, 4, 54]
5	10	160	[35, 20, 7, 20, 7, 6, 5, 2, 7, 11, 2, 7, 11, 9, 6, 5]	[4, 4, 4, 4, 64, 4, 4, 4, 4, 64]
5	10	180	[30, 8, 21, 2, 21, 7, 2, 2, 5, 14, 7, 2, 2, 19, 8, 3, 5, 22]	[6, 6, 6, 6, 66, 6, 6, 6, 6, 66]

## Appendix A

**Proof** **of** **Theorem** **3.** Since *l* is relatively prime to *m*, the integers 0,l,2l,⋯,(m−1)l are distinct modulo *m*. Because the seed codes of length *n* are from *r* different full-order twin-necklace sets with mono-difference, we know that the codeword list in the statement of the theorem do form a cyclic Gray code. Then we will prove that the codewords have single-track property. Let $A^{0},A^{1},\cdots ,A^{m-1}$ be the first m columns of the seed codes, and $B^{0},B^{1},\cdots ,B^{m-1}$ be the last *m* columns of the seed code. The first column of the entire codeword matrix $W^{0}$ is constructed by every *l* columns of $A^{0},A^{1},\cdots ,A^{m-1}$, $W^{0}={\left[A^{0},A^{l},A^{2l},\cdots ,A^{(m-1)l}\right]}^{T}$, where the superscripts are reduced modulo *m*. The basic structure of the codeword list can be regarded as two length *m* necklace ordering combined together, so any column of the codeword list can be represented as:

$$\begin{array}{l} W^{j}={\left[A^{j},A^{j+l},A^{j+2l},\cdots ,A^{j+(m-1)l}\right]}^{T}\\ W^{j+m}={\left[B^{j},B^{j+l},B^{j+2l},\cdots ,B^{j+(m-1)l}\right]}^{T} \end{array}$$

where 0≤j<m and the superscripts are reduced modulo *m*. Since *l* and *m* are relatively prime, for every *j* we have j=il(modm) for some *i*. So $W^{j}$ and $W^{j+m}$ are:

$$\begin{array}{l} W^{j}={\left[A^{il},A^{(i+1)l},A^{(i+2)l},\cdots ,A^{(i+m-1)l}\right]}^{T}\\ W^{j+m}={\left[B^{il},B^{(i+1)l},B^{(i+2)l},\cdots ,B^{(i+m-1)l}\right]}^{T} \end{array}$$

i.e., $W^{j}=E^{il\cdot r}W^{0}$ and $W^{j+m}=E^{il\cdot r}W^{m}$. From the structure of the theorem, we know that $W^{j+m}=E^{t}W^{0}$, so every column of the codeword list is the cyclic shift of $W^{0}$,

$$W^{j}=E^{il\cdot r}W^{0}\;\mathrm{and}\;W^{j+m}=E^{il\cdot r+t}W^{0}$$

Therefore, the codeword list is a STGC. ☐

## Appendix B. Complete Result of Length-6 STGCs

**Table A1:** Complete result of length 6 necklace ordering STGCs.

Period	Generating Sequence	Head Interval
12	[9, 3] [7, 5]	[2, 2, 2, 2, 2, 2]
24	[14, 3, 2, 5] [13, 2, 3, 6] [11, 2, 5, 6] [11, 3, 3, 7] [10, 3, 2, 9] [10, 5, 2, 7] [9, 6, 3, 6]	[4, 4, 4, 4, 4, 4]
36	[16, 3, 2, 2, 3, 10] [16, 3, 2, 5, 3, 7] [16, 4, 3, 2, 2, 9] [15, 2, 3, 5, 3, 8] [15, 2, 3, 8, 3, 5] [15, 2, 2, 3, 4, 10] [15, 2, 2, 9, 4, 4] [14, 2, 5, 8, 2, 5] [14, 2, 3, 4, 4, 9] [14, 3, 5, 9, 2, 3] [14, 3, 4, 7, 3, 5] [14, 3, 2, 3, 5, 9] [11, 2, 8, 5, 2, 8] [11, 2, 3, 10, 7, 3] [11, 3, 8, 9, 2, 3] [10, 3, 2, 8, 9, 4] [9, 2, 8, 3, 4, 10] [13, 2, 2, 5, 4, 10] [13, 3, 4, 7, 2, 7] [8, 2, 7, 4, 4, 11]	[6, 6, 6, 6, 6, 6]
48	[15, 2, 3, 2, 5, 7, 3, 11] [15, 2, 2, 7, 3, 9, 6, 4] [15, 3, 2, 5, 4, 9, 5, 5] [15, 3, 2, 5, 4, 9, 5, 5] [15, 4, 6, 3, 2, 4, 3, 11] [15, 7, 5, 7, 3, 4, 3, 4] [14, 3, 3, 11, 4, 2, 5, 6] [14, 11, 2, 4, 3, 3, 7, 4] [13, 5, 7, 2, 4, 5, 2, 10] [11, 2, 9, 3, 3, 3, 3, 14] [11, 2, 5, 13, 7, 3, 3, 4] [11, 3, 6, 5, 4, 5, 5, 9] [10, 2, 9, 2, 2, 13, 5, 5] [10, 3, 9, 11, 2, 4, 5, 4] [10, 3, 6, 4, 5, 5, 5, 10] [9, 2, 4, 3, 4, 14, 9, 3] [9, 3, 6, 4, 5, 4, 6, 11] [14, 3, 4, 2, 4, 7, 2, 12] [14, 3, 4, 5, 2, 7, 4, 9] [14, 3, 4, 14, 4, 3, 2, 4] [14, 3, 3, 3, 4, 7, 3, 11] [14, 3, 3, 14, 5, 4, 2, 3] [14, 3, 2, 7, 3, 10, 5, 4] [14, 4, 5, 2, 3, 7, 2, 11] [14, 4, 5, 4, 2, 4, 3, 12] [14, 4, 3, 2, 5, 13, 2, 5] [14, 4, 3, 4, 3, 7, 4, 9] [14, 4, 2, 5, 4, 10, 4, 5] [14, 4, 2, 13, 4, 2, 4, 5] [13, 2, 5, 5, 2, 7, 4, 10] [13, 2, 5, 13, 2, 3, 4, 6] [13, 2, 4, 3, 4, 7, 3, 12] [13, 2, 3, 7, 3, 10, 5, 5] [13, 2, 3, 7, 2, 11, 6, 4] [13, 4, 6, 4, 3, 4, 2, 12] [13, 4, 3, 11, 4, 3, 4, 6] [13, 4, 2, 12, 5, 2, 4, 6] [12, 2, 7, 10, 3, 7, 2, 5] [12, 2, 7, 12, 2, 4, 3, 6] [12, 2, 5, 4, 3, 7, 4, 11] [12, 2, 5, 4, 3, 11, 4, 7] [12, 2, 5, 4, 2, 12, 5, 6] [12, 2, 4, 5, 4, 10, 4, 7] [12, 3, 7, 11, 2, 7, 3, 3] [12, 5, 6, 3, 4, 6, 2, 11] [12, 5, 4, 5, 4, 5, 4, 9] [11, 4, 5, 5, 4, 5, 4, 10] [11, 2, 5, 5, 2, 5, 6, 12]	[8, 8, 8, 8, 8, 8]

**Table A2:** Complete result of length 6 self-dual necklace ordering STGCs.

Period	Generating Sequence	Head Interval
12	[6, 6]	[1, 1, 1, 1, 1, 7] and its equivalent head intervals ^a^
36	[11, 2, 5, 11, 2, 5] [10, 4, 4, 10, 4, 4] [8, 2, 8, 8, 2, 8]	[3, 3, 3, 3, 3, 21] and its equivalent head intervals ^b^
60	[13, 3, 6, 2, 6, 13, 3, 6, 2, 6] [13, 4, 7, 2, 4, 13, 4, 7, 2, 4] [12, 2, 4, 8, 4, 12, 2, 4, 8, 4] [11, 2, 6, 3, 8, 11, 2, 6, 3, 8] [11, 3, 9, 4, 3, 11, 3, 9, 4, 3]	[5, 5, 5, 5, 5, 35] and its equivalent head intervals ^c^

^a^ The equivalent head intervals of [1, 1, 1, 1, 1, 7] are [1, 1, 1, 2, 5, 2], [1, 1, 2, 1, 4, 3], [1, 2, 2, 3, 2, 2] and [1, 3, 1, 3, 1, 3]. ^b^ The equivalent head intervals of [3, 3, 3, 3, 3, 21] are [3, 3, 3, 6, 15, 6], [3, 3, 6, 3, 12, 9], [3, 6, 6, 9, 6, 6] and [3, 9, 3, 9, 3, 9]. ^c^ The equivalent head intervals of [5, 5, 5, 5, 5, 35] are [5, 5, 5, 10, 25, 10], [5, 5, 10, 5, 20, 15], [5, 10, 10, 15, 10, 10] and [5, 15, 5, 15, 5, 15].

**Table A3:** Complete result of length 6 twin-necklace ordering STGCs.

Period	Generating Sequence	Head Interval
12	[6, 6]	[1, 3, 1, 3, 1, 3]
24	[14, 4, 2, 4] [12, 2, 4, 6]	[3, 5, 3, 5, 3, 5]
36	[14, 4, 2, 5, 6, 5] [11, 6, 5, 6, 2, 6] [10, 4, 6, 8, 2, 6] [10, 6, 4, 6, 4, 6] [10, 6, 2, 5, 6, 7] [8, 6, 4, 4, 6, 8] [8, 6, 8, 6, 2, 6] [11, 2, 5, 11, 2, 5] [10, 4, 4, 10, 4, 4] [8, 2, 8, 8, 2, 8]	[3, 9, 3, 9, 3, 9]
48	[8, 3, 7, 11, 6, 2, 5, 6] [15, 6, 3, 2, 3, 5, 5, 9] [15, 6, 2, 3, 3, 5, 6, 8]	[4, 12, 4, 12, 4, 12]
	[12, 6, 2, 4, 6, 8, 4, 6] [14, 8, 6, 6, 2, 4, 2, 6] [14, 4, 6, 12, 2, 4, 2, 4]	[3, 13, 3, 13, 3, 13]
60	[12, 2, 4, 6, 4, 8, 6, 4, 4, 10]	[9, 11, 9, 11, 9, 11]
	[13, 3, 6, 2, 6, 13, 3, 6, 2, 6] [13, 4, 7, 2, 4, 13, 4, 7, 2, 4] [12, 2, 4, 8, 4, 12, 2, 4, 8, 4] [11, 2, 6, 3, 8, 11, 2, 6, 3, 8] [11, 3, 9, 4, 3, 11, 3, 9, 4, 3]	[5, 15, 5, 15, 5, 15]

**Table A4:** Complete result of length 6 triplet-necklace ordering STGCs.

Period	Generating Sequence	Head Interval
24	[9, 6, 3, 6]	[2, 2, 8, 2, 2, 8]
48	[12, 6, 5, 2, 5, 5, 2, 11]	[4, 4, 16, 4, 4, 16]
